# 3K3A-Activated Protein C Prevents Microglia Activation, Inhibits NLRP3 Inflammasome and Limits Ocular Inflammation

**DOI:** 10.3390/ijms232214196

**Published:** 2022-11-17

**Authors:** Dahlia Palevski, Gil Ben-David, Yehonatan Weinberger, Rabeei Haj Daood, José A. Fernández, Ivan Budnik, Sarina Levy-Mendelovich, Gili Kenet, Yael Nisgav, Dov Weinberger, John H. Griffin, Tami Livnat

**Affiliations:** 1 Rabin Medical Center, Ophthalmology Department and Laboratory of Eye Research, Felsenstein Medical Research Center, Petah-Tikva 49100, Israel; 2 Sackler Faculty of Medicine, Tel Aviv University, Tel-Aviv 6997801, Israel; 3 Department of Molecular Medicine, The Scripps Research Institute, La Jolla, CA 92037, USA; 4 Sheba Medical Center, The Amalia Biron Thrombosis Research Institute, Tel-Hashomer 52621, Israel

**Keywords:** activated protein C, inflammation, inflammasome, NLRP3, microglia, uveitis

## Abstract

3K3A-Activated Protein C (APC) is a recombinant variant of the physiological anticoagulant APC with pleiotropic cytoprotective properties albeit without the bleeding risks. The anti-inflammatory activities of 3K3A-APC were demonstrated in multiple preclinical injury models, including various neurological disorders. We determined the ability of 3K3A-APC to inhibit ocular inflammation in a murine model of lipopolysaccharide (LPS)-induced uveitis. Leukocyte recruitment, microglia activation, NLRP3 inflammasome and IL-1β levels were assessed using flow cytometry, retinal cryosection histology, retinal flatmount immunohistochemistry and vascular imaging, with and without 3K3A-APC treatment. LPS triggered robust inflammatory cell recruitment in the posterior chamber. The 3K3A-APC treatment significantly decreased leukocyte numbers and inhibited leukocyte extravasation from blood vessels into the retinal parenchyma to a level similar to controls. Resident microglia, which underwent an inflammatory transition following LPS injection, remained quiescent in eyes treated with 3K3A-APC. An inflammation-associated increase in retinal thickness, observed in LPS-injected eyes, was diminished by 3K3A-APC treatment, suggesting its clinical relevancy. Finally, 3K3A-APC treatment inhibited inflammasome activation, determined by lower levels of NLRP3 and its downstream effector IL-1β. Our results highlight the anti-inflammatory properties of 3K3A-APC in ocular inflammation and suggest its potential use as a novel treatment for retinal diseases associated with inflammation.

## 1. Introduction

Activated Protein C (APC) is a physiological anticoagulant derived from its zymogen protein C (PC). Two major APC activities have been described as involving anticoagulant and cell signaling activities [1,2]. Inhibition of inflammatory responses, endothelial barrier protection, and increased cell survival have emerged as key mechanisms underlying the cellular protective effects of APC [1,2,3].

The importance of endogenous PC during or after fetal development of the eye is provided by clinical data from congenital PC deficiency infants diagnosed with various ocular pathologies of the anterior and posterior segments, which shows children with severe PC deficiency are blind at birth [4,5]. 

The potential use of APC for treating ocular pathologies evolved from a pilot prospective clinical trial for central retinal vein occlusion (CRVO) patients [6]. The authors confirmed the safety and efficacy of APC treatment. They found that a single intraocular injection of APC led to the complete resolution of macular edema with no recurrence in 50% of CRVO patients [6]. As the improvement in the reperfusion of areas of retinal nonperfusion was maintained in the long term, with no complications, the authors suggested that intravitreal APC treatment is a safe and potential alternative treatment for CRVO [7]. 

As APC is a natural coagulation inhibitor, bleeding risk may limit its use as a therapeutic agent. Indeed, therapeutic recombinant APC was approved in adult patients to treat severe sepsis [8]. However, therapy was complicated by bleeding events that were considered side effects of APCs’ anticoagulant properties [8]. A recombinant engineered variant of wild-type (wt)-APC, 3K3A-APC (where three Lys residues replace three Ala residues), displays markedly reduced anticoagulant activity. The replacement of the three residues in the 3K3A-APC variant reduces APC’s interactions with the clotting factor Va and diminishes its anticoagulant activity [9]. Importantly, 3K3A-APC sustains its pleiotropic cytoprotective activities and preserves the interactions with its cell receptors, including binding to endothelial protein C receptors (EPCR) and activating protease-activated receptors (PAR)1 and PAR3 [9]. 

Beneficial cytoprotective activities of 3K3A-APC are reported in many disease models of different organs that are manifested as anti-inflammatory, anti-apoptotic, and endothelial and epithelial cell barrier protective activities, as well as regenerative effects [1,2,10]. Furthermore, the multiple neuroprotective actions of 3K3A-APC led to a successful translation from preclinical to phase II clinical studies for acute ischemic stroke [11,12]. 

Studying the effects of 3K3A-APC in the retina, our group confirmed that 3K3A-APC inhibits and regresses choroidal neovascularization (CNV) growth and preserves wt-APC’s protective activities in the retina in a murine model of laser-induced CNV [13,14]. Furthermore, we found that 3K3A-APC and wt-APC significantly reduced vascular endothelial growth factor (VEGF) levels at CNV sites [13]. 

The anti-inflammatory effects of APC and 3K3A-APC have long been appreciated in non-ocular tissues. APC’s broad-spectrum activities include the inhibition of nuclear factor kappa B (NF-*κ*;B) activation, changes in gene expression profiles, the downregulation of adhesion molecules on endothelial cells, reduced leukocyte adhesion and infiltration, the inhibition of neutrophil NETosis, and the cleavage of extracellular histones [1,2,15]. Recent studies suggest that APC and 3K3A-APC can mediate their anti-inflammatory effects via suppressing the NLRP3 inflammasome [15,16]. Currently, however, no data are available regarding the anti-inflammatory effects of 3K3A-APC in ocular pathologies. As accumulating evidence implies that inflammatory activation contributes to tissue damage in many ocular diseases, including uveitis, glaucoma, diabetic retinopathy, and age-related macular degeneration (AMD) [17,18,19], there is a growing need to target ocular inflammation using novel treatment strategies.

Uveitis represents a diverse array of intraocular inflammatory conditions that can be associated with complications from autoimmune diseases, bacterial infections, viral infections and chemical injuries, and it constitutes 10–15% of all cases of blindness [17,20]. 

Endotoxin-induced uveitis (EIU) using lipopolysaccharide (LPS) is an established animal model for ocular inflammation [21,22,23]. In this model, an increase in inflammatory mediators leads to blood retinal barrier (BRB) breakdown, leukocyte influx and retinal edema [22,23]. Hence, it has been seen as a valuable model for non-autoimmune human anterior uveitis and panuveitis, as well as for acute ocular inflammation driven by the innate immune response [24,25,26].

In the current study, we elaborate on our findings regarding the beneficial use of 3K3A-APC as a broad-spectrum cytoprotective molecule in retinal disease. We used the EIU inflammation murine model to demonstrate the anti-inflammatory properties of 3K3A-APC in the eye. 

## 2. Results

### 2.1. LPS-Induced EIU Causes Immune Cell Accumulation in Different Eye Compartments

**Figure 1:**
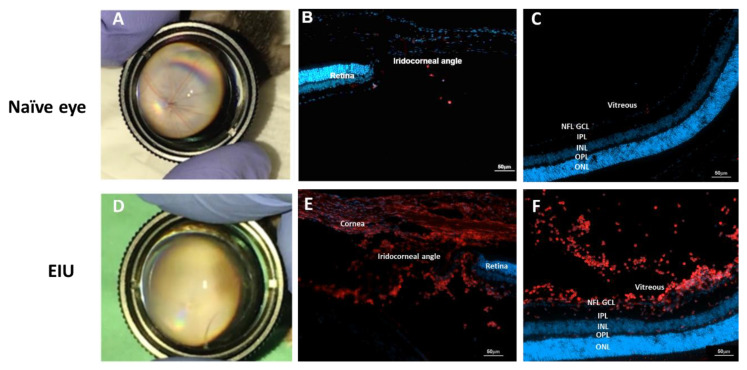
Intravitreal LPS increases inflammatory cell accumulation in the eye. (**A**) Indirect ophthalmoscopy fundus images of naïve eyes demonstrated a normal fundus with clean vitreous. (**B**,**C**) Representative immunofluorescence images of CD11b staining (myeloid cells) in cryosections of iridocorneal angle and retina in naïve eyes, showing minimal CD11b staining in both sections. (**D**) 24 h post-EIU induction, a notable vitreal haze (vitritis) is noticed, partially obscuring the optic disc. Robust CD11b staining is noted in and behind the iridocorneal angle (**E**) and vitreous and retina (**F**) following EIU, indicating a posterior inflammation. Blue—nuclei; Red—CD11b. Scale bar represents 50 µm. EIU—endotoxin-induced uveitis; GCL—ganglion cell layer; INL—inner nuclear layer; IPL—inner plexiform layer; LPS—lipopolysaccharide; NFL—nerve fiber layer; ONL—outer nuclear layer; OPL—outer plexiform layer.

To determine the effect of LPS-induced uveitis in our model, we initially performed an in vivo clinical evaluation of mouse eyes by indirect ophthalmoscopy at several time-points post-LPS injection. In concurrence with previous reports [27], we found the peak of the inflammatory response was 24 h post-EIU induction, assessed by the amount of vitreous haze. Representative fundus images of naïve and LPS-injected eyes 24 h post-EIU induction are presented in Figure 1A,D. We further confirmed the inflammatory reaction in isolated mouse eyes 24 h post-EIU induction by immunofluorescence staining for the myeloid marker CD11b in cryosections of different eye compartments (Figure 1B,C,E,F). Naïve eyes had minimal staining for CD11b. After EIU induction, a notable increase in myeloid cells was detected in the iridocorneal angle, vitreous and retina, indicating a robust inflammatory response in the eye. Clinical examination did not indicate a strong anterior chamber reaction, as we have not seen evidence of hypopyon or marked anterior chamber inflammatory cell accumulation on histology. The majority of the immune reaction, both from clinical examination and histological analysis, was located in the posterior segment of the eye, in the vitreous and retina (Figure 1D,F), defining the EIU model as posterior inflammation.

### 2.2. 3K3A-APC Inhibits Inflammatory Cell Extravasation into the Retinal Parenchyma and Increases Myeloid Retention in Blood Vessels

**Figure 2:**
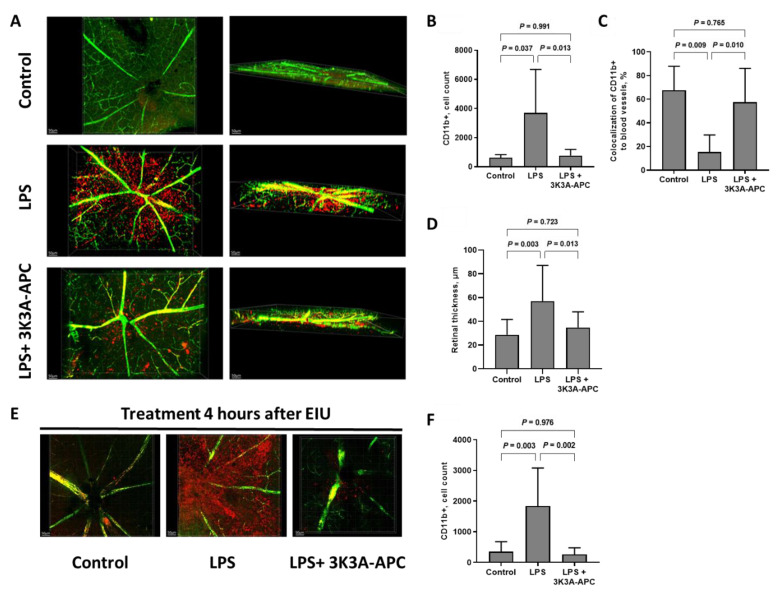
3K3A-APC reduces inflammatory cell numbers and inhibits their extravasation from blood vessels in EIU. (**A**) Representative retinal flatmount images of each treatment group at 24 h with 3K3A-APC pretreatment. Retinal blood vessels were stained using fluorescein isothiocyanate (FITC)-dextran perfusion (green), and inflammatory myeloid cells were stained using anti-CD11b antibody (red). Left panel demonstrates upper view, and right panel represents Z-plane (depth) (scale bar = 50 um). Quantification of total number of CD11b+ cells within the retinal parenchyma (**B**) and co-localization of CD11b+ cells to blood vessels (**C**) in control, LPS or LPS+3K3A-APC treatment shows 3K3A-APC pretreatment significantly reduces total number of CD11b+ cells inside the retina and inhibits their extravasation from retinal blood vessels. (**D**) Retinal thickness is increased following intravitreal LPS injection, an effect attenuated by 3K3A-APC treatment. (**E**) Representative upper view retinal flatmount images of controls, LPS only or LPS+ 3K3A-APC given 4 h after inflammatory induction. (**F**) Quantification of total number of CD11b+ cells within the retinal parenchyma when 3K3A-APC was administered 4 h post-LPS injection. Data are presented as mean ± SD (*n* = 5–9 per group) and were analyzed using one-way ANOVA followed by Tukey’s post hoc test. EIU—endotoxin-induced uveitis; ITV—intravitreal; LPS—lipopolysaccharide.

Mice received an intravitreal (ITV) injection of either vehicle or 3K3A-APC and one hour later LPS was injected ITV to induce experimental uveitis. An in-vivo injection of fluorescein isothiocyanate (FITC)-dextran was performed to fluorescently mark retinal blood vessels 24 h post-EIU induction with and without 3K3A-APC treatment. Immediately after this, retinas were isolated and prepared as flatmounts. Additional immunostaining for CD11b allowed the detection of myeloid cells inside blood vessels and those inside the retinal parenchyma. As expected, almost no CD11b staining existed in eyes not exposed to LPS (controls) (Figure 2A). In eyes exposed to LPS and treated with saline, CD11b staining was demonstrated throughout the entire surface and depth of the retina and was not restricted to blood vessels alone. Quantitative measurements showed that LPS significantly increased the overall number of CD11b+ cells in the retina compared with controls (3968 ± 2984 vs. 629 ± 202 cells; *p* < 0.05, Figure 2B). The pretreatment of 3K3A-APC significantly reduced CD11b+ cell numbers, compared with LPS alone, to a level almost similar to controls (770 ± 418 cells vs. 3968 ± 2984 cells; *p* < 0.05, Figure 2B). When analyzing co-localization of CD11b+ cells to retinal vessels, an increased percentage of myeloid cells retained in blood vessels was detected in 3K3A-APC-treated eyes, compared with LPS alone (58 ± 29% vs. 15 ± 14%; *p* < 0.05), similarly to controls that also demonstrated significantly higher myeloid cell retention in vessels, compared with LPS alone (68 ± 20% vs. 15 ± 14%; *p* < 0.01, Figure 2C). This indicates that the anti-inflammatory effect of 3K3A-APC is associated with both a decrease in overall myeloid cell recruitment to the retina and with decreased extravasation and higher retention within retinal blood vessels.

Retinal thickness is another morphologic marker for inflammation and is used to assess disease activity in humans [28]. Quantitative analysis of blood vessel depth demonstrated a significant increase in vessel volume in the LPS group, compared with controls (57 ± 30 µm vs. 29 ± 13 µm; *p* < 0.05, Figure 2D), indicating an increase in retinal thickness in eyes treated with LPS. Treatment with 3K3A-APC, however, resulted in a much smaller increase in retinal thickness, compared with LPS only (35 ± 13 µm vs. 57 ± 30 µm; *p* < 0.05, Figure 2D), indicating a successful reduction in retinal inflammation, which could have clinical relevancy. To evaluate the potential clinical use of 3K3A-APC, we attempted to simulate the clinical situation wherein anti-inflammatory treatment is given to patients when retinal inflammation already exists. Hence, EIU was induced with ITV LPS, and four hours later eyes were treated with either 3K3A-APC or vehicle control. Retinal flatmounts were prepared as described above. In eyes treated with 3K3A-APC four hours post-LPS, a dramatic reduction in CD11b staining was visible, indicating that 3K3A-APC treatment following existing inflammation still succeeded in reducing myeloid cell numbers in the retina (Figure 2E). Whereas CD11b+ cell numbers markedly increased after LPS injection (347 ± 327 cells vs. 1837 ± 1240 cells in controls and LPS, respectively; *p* = 0.003, Figure 2F), 3K3A-APC treatment significantly reduced their numbers, to a level similar to that in the controls (262 ± 217 cells vs. 1837 ± 1240 cells; *p*= 0.002, Figure 2F).

### 2.3. 3K3A-APC Reduces Recruitment of Leukocytes without Altering the Type of Inflammatory Cell Subpopulations after EIU Induction

**Figure 3:**
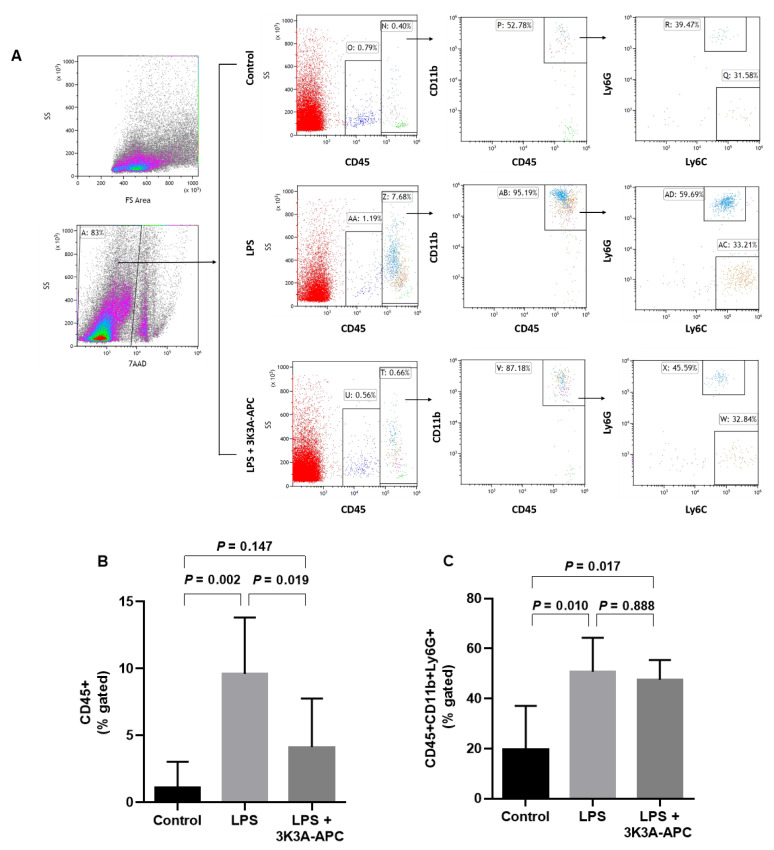
Flow cytometry analysis reveals 3K3A-APC markedly reduces infiltrating leukocytes in EIU. (**A**) A total of 24 h after intravitreal injections, single cell preparations of isolated vitreous and retinas were analyzed using flow cytometry in all treatment groups. Left panel indicates gating strategy used, which excludes debris and dead cells using 7AAD. Levels of CD45+ leukocytes are scarce in control retinas. There is a notable increase in inflammatory CD45+ leukocytes in EIU, compared with controls and with 3K3A-APC-treated eyes. The subpopulation of CD45+CD11B+Ly6G+ cells (neutrophils) is highly increased by LPS injection, while CD45+CD11b+Ly6C+ (macrophages) percentages are relatively stable in all groups. 3K3A-APC reduces levels CD45+ cells in the retina to a level comparable to controls. (**B**) Statistical analysis of CD45+ levels demonstrates a significant increase after ITV LPS, which is inhibited by 3K3A-APC treatment. (**C**) The relative percent of CD45+CD11b+Ly6G+ cells is significantly increased after ITV LPS, but not significantly changed by 3K3A-APC treatment. The data are presented as mean ± SD and analyzed using mixed-effects models followed by Tukey’s post hoc test (*n* = 6–7 pooled retinas per group, with a total of 5 experiments repeated). EIU—endotoxin-induced uveitis; ITV—intravitreal; LPS—lipopolysaccharide.

To assess the anti-inflammatory potential of 3K3A-APC, we aimed to determine its effect on leukocyte recruitment and innate immune myeloid cell subpopulations, such as macrophages and neutrophils in the retina following EIU using flow cytometry. Flow cytometry gating strategy used is presented in Figure 3A. Control eyes were largely devoid of CD45+ (leukocyte) cells apart from a population of CD45lowCD11blow cells that were Ly6G- and Ly6C-, representing resident retinal microglia [27]. At 24 h following EIU induction, flow cytometry revealed a robust inflammatory response in the LPS-injected eyes compared with controls, determined by increased levels of CD45+ cells from all sampled cells (1.11 ± 1.94% in control vs. 9.6 ± 4.2% in EIU, *p* = 0.002) and an increased fraction of CD45+CD11b+ (myeloid) cells (63 ± 10% in control vs. 95 ± 3% out of all leukocytes in EIU, *p* < 0.001) (Figure 3A,B). The main effector cells 24 h post-EIU were CD45+CD11b+Ly6G+ cells, representing neutrophils, which were significantly increased following LPS injection, compared with controls (Figure 3A,C) (20 ± 17% vs. 51 ± 13% out of sampled myeloid cells in controls and EIU, respectively; *p* = 0.01). Treatment with 3K3A-APC significantly reduced CD45+ percentage, compared with untreated EIU eyes (4.1 ± 3.6% vs. 9.6 ± 4.2%, respectively, *p* = 0.019) (Figure 3A,B). While 3K3A-APC dramatically decreased the infiltration of leukocytes to the retina after inflammatory induction, the relative percent of CD45+CD11b+Ly6G+ from sampled leukocytes was not significantly changed (Figure 3C), indicating that 3K3A-APC reduces an overall recruitment of leukocytes without altering the type of inflammatory reaction, which is mainly neutrophilic. 

### 2.4. 3K3A-APC Affects the Local Immune Environment by Inhibiting Resident Microglia Activation

**Figure 4:**
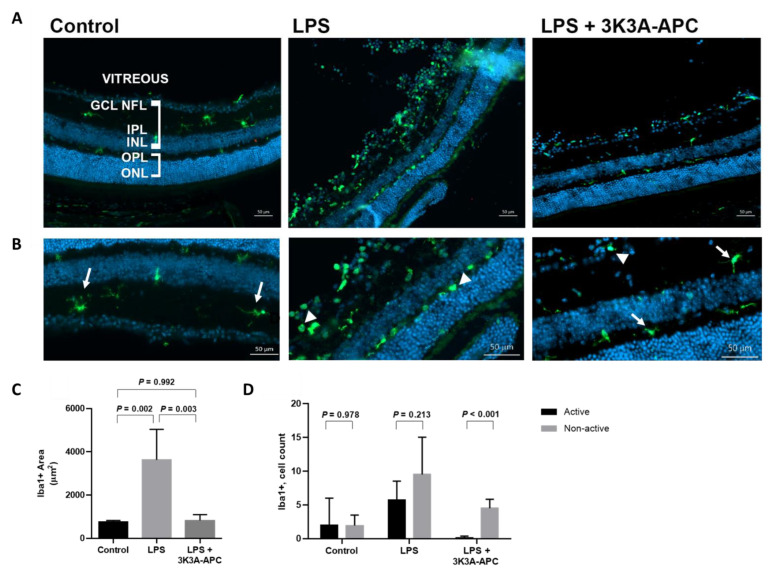
3K3A-APC inhibits microglia cell accumulation and activation after EIU. (**A**) Retinal cryosection images, 24 h after ITV injections. Microglial cells were stained using Iba1, a specific marker for microglia. Panel (**A**) (left) demonstrates scarce microglia mainly in the inner retina (GCL, IPL), which has a ramified morphology, indicating a non-inflammatory state in control eyes. ITV-LPS injection increases the total amount of Iba1-positive cells and their accumulation in the outer retinal layers and induces an inflammatory amoeboid shape (middle). With 3K3A-APC treatment (right), a reduction in Iba1-positive staining is noted and cells resume a more ramified morphology. Scale bar represents 50 µm. (**B**) Magnification of the corresponding cryosections, allowing a clearer distinction between ramified (arrows) and amoeboid (arrowheads) microglia. Scale bar represents 50 µm. Blue—cell nuclei; Green—Iba1. (**C**) Iba1-positive area was calculated using values of 4 microscopic fields (2 fields × 2 slides), which were averaged and used as raw data for further analysis. A significant increase in Iba1 area after LPS injection is noted, an effect prevented in 3K3A-APC-treated eyes. Data are presented as mean ± SD and were analyzed using one-way between-subjects ANOVA followed by Tukey’s post hoc test (*n* = 4–5 per group). (**D**) 3K3A-APC decreases the number of Iba1-positive cells, both active and quiescent, in the outer retina. Compared with LPS only, EIU treated with 3K3A-APC significantly inhibited the number of activated microglial cells, as assessed by an ameboid shape. Data are presented as mean ± SD and were analyzed using unpaired two-tailed Student’s t-test (*n* = 4–5 per group). EIU—endotoxin-induced uveitis; GCL—ganglion cell layer; INL—inner nuclear layer; IPL—inner plexiform layer; ITV—intravitreal; LPS—lipopolysaccharide; NFL—nerve fiber layer; ONL—outer nuclear layer; OPL—outer plexiform layer.

We used the microglia-specific marker ionized calcium-binding adapter molecule 1 (Iba1) to detect their involvement in LPS-induced inflammation and response to 3K3A-APC treatment. We assessed the amount, morphology and anatomical location of microglial cells in the retina using confocal imaging of retinal cryosections. Representative images of control-, LPS- and LPS+3K3A-APC-treated eyes are presented in Figure 4. As expected, only a few microglial cells were detected in control eyes. Without inflammatory stimulation, microglia were located mainly in the inner retina and had a ramified morphology, consistent with a non-inflammatory state [29]. A dramatic increase in Iba1 staining was noted in EIU eyes. Notably, the appearance of microglial cells at the outer layers of the retina presented an inflammatory amoeboid shape, indicating an activated phenotype, whereas 3K3A-APC treatment restricted both the increase in microglial amount and their activation as determined by morphological shape (Figure 4A,B). Quantitative analysis of microglia staining area revealed that LPS induced a statistically significant increase in Iba1 positive area, while 3K3A-APC treatment significantly prevented this increase (Figure 4C). Moreover, we counted the total number of microglia cells at the outer retinal segments (outer nuclear and outer plexiform layers, where microglia cells do not normally reside under physiological conditions). Whereas LPS increased total Iba1+ cell numbers, compared to controls (15.25 ± 3.38 cells vs. 3.94 ± 3.06 cells, respectively; *p* < 0.001), the addition of 3K3A-APC treatment resulted in a significantly lower number of microglial cells in the outer retina (4.63 ± 1.45 cells vs. 15.25 ± 3.38 cells; *p* < 0.001). Next, we determined microglia activation state by their morphology as activated (amoeboid) and non-activated (ramified). In eyes treated only with vehicle control or LPS, there was no difference between the numbers of activated and non-activated cells. However, eyes treated with 3K3A-APC demonstrated a significantly lower number of activated microglial cells, compared with the non-active subpopulation (Figure 4D). These data indicate that 3K3A-APC not only reduces microglial cell accumulation in the eye but also inhibits an activated microglial state.

### 2.5. 3K3A-APC Reduces NLRP3 and Il1β Levels after EIU Induction

**Figure 5:**
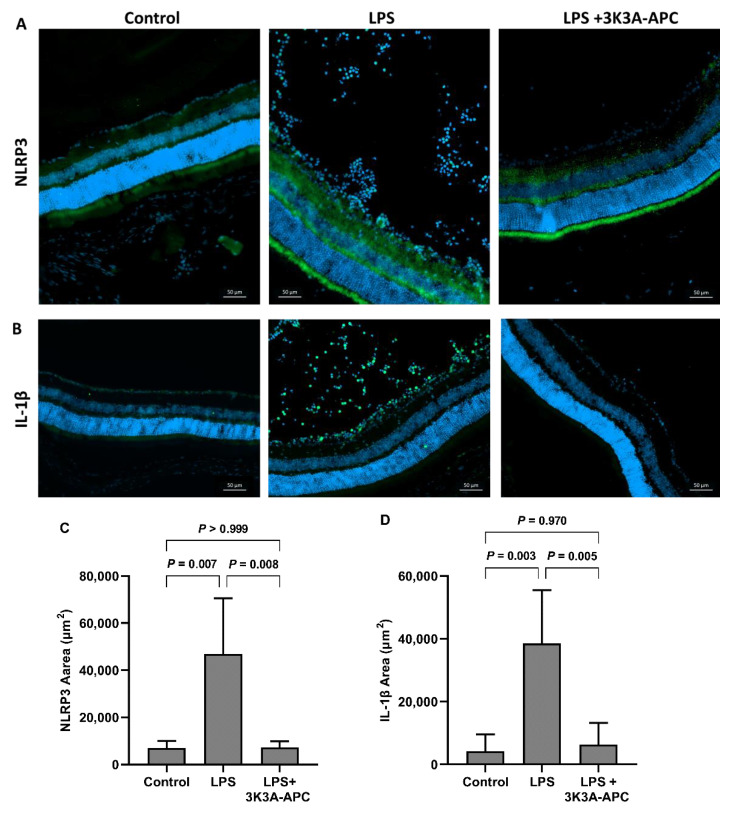
3K3A-APC Reduces NLRP3 and Il1β Levels after EIU Induction. Retinal cryosections were stained using direct antibodies against NLRP3 and IL1-β. Panel (**A**) demonstrates strong NLRP3 staining in the photoreceptor, outer plexiform and ganglion cell layers, which is attenuated following 3K3A-APC injection. Panel (**B**) shows abundant Il-1β+ cells in the vitreous and inner retinal layers following LPS injection, while eyes treated with 3K3A-APC demonstrate weak staining in the inner retinal layers. Scale bar represents 50 µm. Blue—cell nuclei; Green—NLRP3/IL1β. Positive staining areas were calculated using values of 4 microscopic fields (2 fields × 2 slides), which were averaged and used as raw data for further analysis. Data are presented as mean ± SD and analyzed using one-way ANOVA followed by Tukey’s post hoc test (*n* = 4–6 per group). A significant increase in NLRP3 (**C**) and IL1-β (**D**) area after LPS injection is noted, an effect prevented in 3K3A-APC-treated eyes. EIU—endotoxin-induced uveitis; IL-1β—Interleukin-1beta; LPS—lipopolysaccharide; NLRP3—NLR family pyrin domain containing 3.

The NLRP3 inflammasome is a crucial component of the innate immune response, whose activation ultimately results in the release of the pro-inflammatory cytokines interleukin (IL)-1β and IL-18 [13]. As APC has been shown to inhibit murine NLRP3 inflammasome in injury models, such as cardiac ischemia-reperfusion injury [14] and ischemic white matter stroke [29], we aimed to determine whether 3K3A-APC treatment is associated with a decrease in NLRP3 activation in the retina as well. A total of 24 hours post-EIU induction, retinal cryosections were prepared and stained for NLRP3 and IL-1β. Representative images (Figure 5A,B) demonstrate minimal staining for NLRP3 and IL-1β in the control eye. The marked staining of NLRP3 in all retina layers was detected upon LPS injection, which was attenuated after 3K3A-APC treatment (Figure 5A). Compared with control eyes, an increase in IL-1β positive cells was observed after EIU with marked expression in the vitreous cavity. IL-1β increase was prevented in 3K3A-APC-treated eyes, concurrent with NLRP3 reduction (Figure 5B). Quantitative analysis of NLRP3- and IL-1β-positive areas (Figure 5C,D) confirmed the statistical significance of NLRP3/IL-1β elevation following EIU induction that was almost entirely prevented by 3K3A-APC treatment.

## 3. Discussion

APC is a serine protease with several distinguishable biochemical activities. APC exerts anticoagulant activity by inactivating factor Va and VIIIa, and it exerts cytoprotective and anti-inflammatory activities primarily through interaction with EPCR and PAR1 and PAR3 [1,2]. To bypass its anticoagulant properties, Mosnier et al. replaced three Lys residues with Ala residues in the wt-APC molecule, altering the factor Va-binding exosites without modifying the exosites that recognize and bind to PAR1, producing the 3K3A-APC signaling-selective variant, which lacks most of its anticoagulant activity [9]. The neuroprotective, endothelial-barrier-protective and anti-inflammatory properties of 3K3A-APC were demonstrated in multiple preclinical animal studies [1,2,11,30]. In particular, models of neurological injuries have shown that 3K3A-APC has neuroprotective effects manifesting as protection of blood–brain barrier (BBB) function, inhibition of neuroinflammation, inhibition of neuronal apoptosis, and regenerative effects targeting neuronal stem cells, highlighting the pleiotropic cytoprotective functions of 3K3A-APC in the brain and in neurodegenerative diseases [1,11,31]. 

Recently, the multicenter, randomized phase 2 RHAPSODY trial successfully established the safety of 3K3A-APC when administered following alteplase, mechanical thrombectomy, or both in patients with moderate to severe acute ischemic stroke [12]. 

To the best of our knowledge, our group is the only one that has evaluated the use of 3K3A-APC for the potential treatment of ocular pathologies [13]. We previously demonstrated that 3K3A-APC treatment inhibited and regressed CNV growth and retained wt-APC’s protective activities in the retina, in the laser-induced CNV murine model [13,14]. Furthermore, we found that 3K3A-APC significantly reduced vascular endothelial growth factor (VEGF) levels at CNV sites [13]. 

The anti-inflammatory effects of APC and 3K3A-APC have long been appreciated in non-ocular tissues [1,2,15]. Nonetheless, no data have been published regarding the anti-inflammatory activity of 3K3A-APC in the eye.

In the current study, we used the EIU model to elaborate on the anti-inflammatory effects of 3K3A-APC in the eye. The EIU model is extensively used as an innate immune-driven uveitis model for the timely investigation of novel therapeutic agents in the eye [23,24]. Twenty-four hours following intravitreal injection of the endotoxin (LPS), we observed a robust innate immune response in the retina and vitreous, as well as in the anterior chamber of the eye (Figure 1), that was accompanied by an increase in retinal thickness (Figure 2D). Retinal thickness is a sensitive morphologic marker of the degree of inflammation and is used clinically to assess posterior uveitis activity in humans [28]. The observation that treatment with 3K3A-APC limited the increase in retinal thickness induced by inflammation points towards 3K3A-APC’s anti-inflammatory potential. 

Next, we evaluated the impact of 3K3A-APC treatment on inflammatory cell recruitment and extravasation from blood vessels into the retina. treatment with 3K3A-APC led to a significant decrease in leukocyte migration into the retina, seen both by histological analysis of the myeloid cell marker CD11b (Figure 2) and quantified by flow cytometry (Figure 3). Moreover, the inhibition of immune cell infiltration into the retina, even when administered four hours after LPS-induced inflammation (Figure 2E,F), highlights 3K3A-APC’s potential clinical use.

The predominant immune cells involved in our model were neutrophils, followed by macrophages, which together comprised more than 90% of myeloid cells, concurrent with published data on the inflammatory reaction in EIU [27]. We have not assessed other myeloid subpopulations, such as dendritic cells or eosinophils, as they were shown to be virtually non-existent in similar models of EIU [24,27]. We did not detect a preferential inhibition of neutrophil over macrophage chemotaxis, as both subpopulations were similarly affected by 3K3A-APC’s inhibitory actions in the retina (Figure 3). However, as the inflammation induced here was assessed 24 h after endotoxin injection, it is not surprising that the main effector cells were still neutrophilic, even with 3K3A-APC’s anti-inflammatory effect. 

The inhibitory effect of 3K3A-APC on leukocytes in our study also extended to extravasation. We show that not only does 3K3A-APC inhibit leukocyte migration to the retina, but that it also limits myeloid cell extravasation, as demonstrated by decreased CD11b+ cell staining in the retinal parenchyma and increased co-localization to blood vessel walls (Figure 2A,C). We hypothesize that 3K3A-APC’s barrier protective properties could be responsible, at least partially, for the decreased inflammatory cell extravasation seen in our study. The BRB separates the systemic circulation from the retina. It is composed of the inner BRB, formed by the microvasculature of the inner retina, and the outer-BRB retinal pigment epithelium (RPE), which separates the fenestrated choriocapillaris from the retina [32]. The BRBs protect the neural retina and maintain retinal homeostasis, and their function is fundamental for proper retinal function and vision. Disruption of the BRB is a crucial step in ocular pathogenesis and its breakdown can result in edema formation, a hallmark feature of many retinal diseases [32]. Barrier-stabilizing properties of APC were demonstrated in a wide range of animal models and cells, including the outer barrier of the retina, RPE [1,14,33]. Moreover, a clinical trial in CRVO patients, treated with a single injection of wt-APC, confirmed the anti-leakage properties of APC in the retina [6]. Thus, we suggest that APC’s potent ability to stabilize barriers could be part of the mechanism behind the significantly decreased leukocyte extravasation seen in our study. Indeed, APC’s effects on endothelial barriers are considered part of its anti-inflammatory actions [33]. Additionally, APC is known to downregulate the expression of key adhesion molecules, such as ICAM, on the surface of injured endothelium and in murine models of focal ischemic stroke [34], which adds to APC’s inhibitory effects on leukocyte ability to cross blood barriers.

Microglia are the resident immune cells in the central nervous system and retina. In the retina, they reside mainly in the inner retina, where they lay in a quiescent state, monitoring their environment. Under physiological conditions, microglia assume a quiescent state, characterized by a ramified morphology with elongated processes [35]. In response to injury, microglia assume an activated, amoeboid shape and migrate to the site of injury to participate in the inflammatory response [29]. While a necessary immune regulator, unchecked activated microglia can have deleterious effects on their microenvironment through the secretion of inflammatory mediators.. Activated microglia may kill degenerated neurons and photoreceptors through phagocytosis and exacerbate retinal injury and vision loss by producing multiple proinflammatory mediators [25,36,37]. Our data show a considerable increase in the area positive for microglial cells 24 h after ITV-LPS injection. Furthermore, LPS increased microglia cells number at the outer layers, where they do not normally reside under physiological conditions. However, 3K3A-APC treatment limited the total amount of microglial cells and their presence in the outer layers of the retina. The use of 3K3A-APC also inhibited the transformation of the microglia cells into an inflammatory, activated amoeboid shape (Figure 4D). Of note, as there is currently no unique cell surface marker to fully distinguish active microglia cells from resting cells nearby, we have combined specific microglial Iba1 immunostaining with morphological assessment of their processes to allow us to better characterize activated vs. quiescent microglia. Still, it is possible that we have also included resting, non-activated microglial cells in our analysis.

Our findings are consistent with previous data, showing that 3K3A-APC almost completely suppresses the migration of microglia to the area of lesion in ischemic injury of the corpus callosum in middle-aged mice [31], significantly lowers numbers of microglia in the hippocampus and cortex tissue in a mouse model of Alzheimer’s disease [38], and suppresses microglial activation in an experimental autoimmune encephalomyelitis mouse model of multiple sclerosis (MS) [10].

Microglia have an increasingly recognized role in various retinal pathologies, including uveitis [39], AMD [40], diabetic retinopathy [41] and optic nerve injury [29]. The ability of 3K3A-APC to inhibit microglial recruitment, extravasation, activation and translocation from the inner to the outer retina is critical for its therapeutic potential to limit the progression of inflammatory damage in neurodegenerative ocular diseases with vision loss. 

Recent studies suggest that APC and 3K3A-APC can mediate anti-inflammatory effects via the suppression of the NLRP3 inflammasome [15,16,42]. Inflammasomes function as intracellular sensors of microbial pathogens and foreign and host-derived danger signals. Upon activation, they induce an innate immune response by secreting the inflammatory cytokines IL-1β and IL-18, generated from their pro-IL precursors by caspase-1, which is generated by the activation of procaspase-1 by the inflammasome. The NLRP3 inflammasome is constitutively expressed in various parts of the eye, including the RPE, retinal microglia, Müller cells, astrocytes, conjunctiva, trabecular meshwork and corneal epithelial cells [43]. Accumulating evidence implies that inflammasome activation contributes to tissue damage in various ocular diseases, including glaucoma, diabetic retinopathy and AMD [20], and aberrant activation of the NLRP3 inflammasome was demonstrated in uveitis patients and murine models of uveitis [44]. Thus, the inflammasome became a novel molecular target in retinal disease. Previous studies have shown that wt-APC can inhibit inflammasome activation in models of cardiac and renal ischemia-reperfusion injury via mTORC1 inhibition, alleviating injury [16]. In the same study, 3K3A-APC was also shown to restrict myocardial NLRP3 and IL-1β expression and was dependent on PAR1 signaling [16]. In accordance with these studies, we show here that in the retina, 3K3A-APC also acts via inflammasome inhibition. We demonstrate that in the inflamed retina, 3K3A-APC decreased both inflammasome priming (demonstrated by decreased NLRP3 amount) and its activation through decreased IL-1β levels (Figure 5). With recent developments in anti-inflammasome therapies, some of which are even examined in clinical trials for diseases, such as AMD [17], our results add 3K3A-APC to the increasing list of NLRP3 inhibitors beneficial in ocular pathologies. Moreover, as neutrophils and retinal microglia-driven NLRP3 activation and IL-1β production play an essential role in murine bacterial endophthalmitis [45], 3K3A-APC treatment should be considered as a potential treatment for devastating, uncontrolled endophthalmitis.

The retina, albeit its peripheral location, is part of the central nervous system (CNS). The BRB and the brain–blood barriers share functional and structural similarities and are derived from the developing neural tube [46]. Notably, the strong link between retinal and CNS pathologies is supported by evidence that ocular alterations in various neurodegenerative diseases of the CNS sometimes precede central symptoms [46]. The cell signaling of 3K3A-APC mediated by EPCR, PAR1 and PAR3 is central to its cellular activities in the CNS, and such signaling initiates pleiotropic actions on neurons, glia and cerebral vascular cells [1,30]. EPCR is strongly expressed in human retinal endothelial cells [47]. PAR1 expression was reported in retinal ganglion cells (RGC), Müller glial, rod photoreceptors (but not in cones) and the inner nuclear layer [48,49,50]. Although currently no data are available, it is appealing to hypothesize that 3K3A-APC initiates its cytoprotective signaling in the retina through its most common receptors, including EPCR, PAR1 and PAR3.

Finally, the triad consisting of inflammation, BRB disruption and neuronal injury characterizes most retinal pathologies impairing vision, such as uveitis, AMD and diabetic retinopathy. Currently, it is widely accepted that single-action, single-target agents are unlikely to treat these disorders fully. Hence, there is an unmet need for new pleiotropic agents that will impede inflammation, tighten the BRB and induce neuroprotection, thus, preserving retinal function and vision in the long term. Our data along with 3K3A-APC’s clinical safety profile [12] and well-established, anti-inflammatory blood barrier protection and neuroprotective activities [1,2] suggests that 3K3A-APC should be considered as a new therapeutic option for retinal disease.

## 4. Materials and Methods

### 4.1. Endotoxin-Induced Uveitis Animal Model

The 8–10-week-old male C57BL/6J mice weighing 19 to 25 g were purchased from Envigo RMS (Jerusalem, Israel). All animal experiments were performed according to the ARVO statement’s guidelines for the Use of Animals in Ophthalmic and Visual Research and the approval of the Institutional Animal Care and Use Committee at Rabin Medical Center. Murine recombinant 3K3A-APC (0.82 µg/µL, Griffin lab, San Diego, CA, USA) was prepared as previously described [51,52]. Intravitreal 3K3A-APC concentration was chosen based on previously performed dose-dependent analysis of intravitreal injection of wt-APC [14], and a following study comparing ITV injection of wt-APC and 3K3A-APC [13].

Mice were randomized to receive 1 μL ITV injection of either vehicle or 3K3A-APC. A total of 1 µL of 250 ng lipopolysaccharide (LPS) from *Escherichia coli* (Sigma-Aldrich, St. Louis, MO, USA) diluted in saline was injected ITV to induce experimental uveitis, as previously described [53,54]. All injections were performed under an operating microscope (Zeiss Opmi 6S Microscope; Carl Zeiss Microscopy GmbH, Oberkochen, Germany) using a microsyringe (33-gauge; Hamilton, Reno, NV, USA). Animals were anesthetized with intraperitoneal injection of ketamine (100 mg/kg) and xylazine (10 mg/kg). For all animal experiments, animal allocation to treatments was randomized, and each experiment was repeated 2–3 times. 

### 4.2. Vascular Imaging and Flatmount Immunostaining

Mice received either a pretreatment of 1 μL ITV 3K3A-APC or saline one hour before LPS was injected, or the treatment was administered 4 h after LPS injection. A total of 24 h post-EIU induction (peak of inflammation), mice were anesthetized, and 0.1 mL of 25 mg/mL Fluorescein Isothiocyanate (FITC) dextran conjugate (MW 500k, Sigma-Aldrich, St. Louis, MO, USA) was injected into the left ventricle of the mouse heart. Five minutes later, mice were sacrificed, and a flatmount specimen of sensory retina was separated from the eyecup and flattened on slides [14]. Flatmount specimens were fixed in 4% Para Formaldehyde (PFA) for 10 min. The slides were covered with anti-fade reagent (Invitrogen, Waltham, MA, USA). For anti-CD11b immunostaining, flat-fixed sensory retina slides were incubated in phosphate-buffered saline (PBS)-Triton X100 0.5% solution at 4 °C overnight and later blocked for 2 h at room temperature (RT) in 5% normal donkey serum (NDS; Sigma Aldrich, St. Louis, MO, USA). Slides were incubated with rat anti-mouse CD11b antibody (1:200; Abcam, Cambridge, UK) at 4 °C overnight. Alexa Fluor 568 conjugated goat anti-rat IgG (1:100; Invitrogen, Waltham, MA, USA) was used for the secondary antibodies. A specimen incubated with non-immune serum was used as staining control. Images of 3 dimensional (3D) projections were captured, as previously described [14], using the Leica TCS SP8 confocal microscope (Leica Biosystems, Wetzlar, Germany), and the volume and the depth of FITC dextran staining was measured using the Imaris x64 7.1.1 software (Oxford Instruments, High Wycombe, UK). For retinal thickness measurement, the Z axis of two different images (one spanning the optic nerve and the other at a distal end) were measured at the center of each image and averaged.

### 4.3. Cryosection Histology and Immunofluorescence Staining

Mice received 1 μL ITV injection of either vehicle or 3K3A-APC, and one hour later LPS was injected ITV. At 24 h post-EIU, mice were sacrificed and eyes were removed, punched with a 30 g needle and fixed in 4% PFA for 2 h at RT. Eyes were washed with increasing concentrations of sucrose in PBS and incubated with a final concentration of 30% sucrose overnight at 4 °C. Eyes were then embedded in OCT compound (Sakura Finetek, Tokyo, Japan) on dry ice and kept at −80 °C. Serial sections of 10 µm thickness were cut using a cryostat (Leica Biosystems, Wetzlar, Germany). Sequential cryosections of every fifth section of each eye were blocked with 10% NDS for 1 h at RT and then incubated with rat anti-mouse CD11b (1:200, Abcam, Cambridge, UK), rabbit anti-mouse Iba1 (WAKO, Hiroshima, Japan) (2 µg), rabbit anti-human IL1β (1:25, Abcam, Cambridge, UK) or rabbit anti-mouse NLRP3 (1:200, Abcam, Cambridge, UK) at 4 °C overnight. The next day, sections were incubated with Alexa Fluor 568 conjugated goat anti-rat IgG or Alexa Fluor 488 conjugated donkey anti-rabbit secondary antibody (1:100, Invitrogen, Waltham, MA, USA). Nuclei were counterstained with DAPI (Nucblue fixed cell stain, Molecular Probes, Eugene, OR, USA). Images were captured using a fluorescence microscope (Axio Imager.Z2, Carl Zeiss Microscopy GmbH, Oberkochen, Germany). 

### 4.4. Retinal Dissociation and Flow Cytometry Analysis

Mice received 1 μL ITV injection of either vehicle or 3K3A-APC, and one hour later LPS was ITV injected. A total of 6–7 retinas were pooled to obtain > 1 million cells for flow cytometry. Retinas were isolated, as previously described [24]. Briefly, retinas were entirely removed from the intact eye and placed in 1 mL of ice-cold serum-free RPMI (Biological Industries, Beit Haemek, Israel) and dissected into small pieces. Retinas were further enzymatically digested in 1 mg/mL Collagenase B (Roche, Basel, Switzerland) and 0.5 mg/mL DNASe 1 (Sigma-Aldrich, St. Louis, MO, USA) for three 10-min cycles in a rotating water bath warmed to 37 °C. The cell suspension was filtered through a 70 µM mesh to remove debris, centrifuged for 5 min (400× *g*, 4 °C) and the pellet was re-suspended and counted for live cells using Trypan Blue (Sigma-Aldrich, St. Louis, MO, USA). Cells were incubated with Fc-block (BioLegend, San Diego, CA, USA) followed by primary conjugated antibody staining. For immune cell markers, the following antibodies were used—Allophycocyanin-conjugated CD45, Phycoerythrin (PE)-CD11b, PE/Cyanine 7 (Cy7)-Ly6C and Brilliant Violet (BV)421—Ly6G (BioLegend, San Diego, CA, USA), according to manufacturer’s protocol. Matched isotype controls were used in a similar manner. Labeled cells from each sample were acquired and analyzed using the Gallios 10-channel flow cytometer and Kaluza Analysis Software (Beckman Coulter, Brea, CA, USA).

### 4.5. Statistical Analysis

Statistical analysis was performed using GraphPad Prism Version 9.3.1 for Windows (GraphPad Software, San Diego, CA, USA). In microscopic imaging studies, the values of four microscopic fields (two slides for an eye, two microscopic fields for a slide) obtained from an eye were averaged into one value and used as raw data for further analysis. The data were presented as mean ± standard deviation (SD) and analyzed using one-way ANOVA followed by Tukey’s post hoc test or unpaired two-tailed Student’s *t*-test, as indicated. In flow cytometry studies, the data were presented as mean ± SD and, to account for inter-experimental variability, analyzed using mixed-effects models, with treatment as a between-subjects factor and experiment as a within-subjects factor, followed by Tukey’s post hoc test. Differences were considered statistically significant if the *p* value was less than 0.05.

## 5. Conclusions

Herein we show that the novel cytoprotective molecule 3K3A-APC can limit the development of ocular inflammation, restrict the innate immune response in the eye, inhibit microglia activation and reduce NLRP3/IL-1β levels. Much remains to be understood regarding the mode of action of 3K3A-APC in the retina. However, the current and former studies [13,14] suggest that 3K3A-APC is a promising pleiotropic agent that may serve as an innovative therapeutic approach for a retinal disease whose underlying pathology is multifactorial. Future preclinical work is needed to set the stage for clinical trials employing 3K3A-APC as therapy for retinal pathologies. 

## Data Availability

Data are available from T.L. upon request.
